# Reconstruction of Multiplex Networks with Correlated Layers

**DOI:** 10.3390/e28040411

**Published:** 2026-04-04

**Authors:** Valerio Gemmetto, Diego Garlaschelli

**Affiliations:** 1 Instituut-Lorentz for Theoretical Physics, University of Leiden, Niels Bohrweg 2, 2333 CA Leiden, The Netherlands; 2 IMT School for Advanced Studies, Piazza San Francesco 19, 55100 Lucca, Italy

**Keywords:** network reconstruction, multiplex networks, world trade web

## Abstract

In many situations, the complete microscopic structure of a network is empirically inaccessible and has to be inferred from aggregate information using some probabilistic model. While several network reconstruction methods have been developed in the case of single-layer networks where nodes can be connected only by one type of link, the problem is still largely unexplored in the case of multiplex networks where several interdependent layers, each representing a distinct mode of connection, coexist. Even the most advanced network reconstruction techniques, when applied to each layer separately, may fail in replicating the inter-layer dependencies embodying the essence of multiplex networks. Here we develop a methodology to reconstruct a class of correlated multiplexes which includes, as a specific example that we study in detail, the multiplex network of international trade in different products. Our method starts from virtually any reconstruction model that successfully reproduces a set of desired marginal properties of each layer separately, i.e., node strengths and/or node degrees. It then introduces the minimal dependency structure required to replicate an additional set of higher-order properties, namely the portion of each node’s degree and each node’s strength that is shared and/or reciprocated across pairs of layers. These properties are found to provide empirically robust measures of inter-layer coupling, allowing for an accurate reconstruction of the world trade multiplex network. Our method allows for joint multi-layer connection probabilities to be reliably reconstructed from marginal ones, effectively bridging the gap between single-layer information and global multiplex properties.

## 1. Introduction

Over the last twenty-five years, the study of complex networks has deepened our understanding of several real-world complex systems, ranging from infrastructures to biological, social and economic systems [1,2,3]. Indeed, diverse systems share a common abstract representation in terms of nodes connected by links, i.e., in terms of graphs or networks. However, a single-network representation is often not enough to fully capture the whole complexity of real-world systems [4]. For instance, the presence of different airline companies significantly affects the properties of the air transportation system [5,6]. Similarly, the human body can be thought of as a set of interdependent networks where several complex physiological systems, e.g., the nervous and the cardiovascular ones, constantly interact [7].

For this reason, the concepts of multiplex and interdependent networks have been developed. In a multiplex network, a given set of nodes is connected through different modes of interactions. The system can be equivalently represented as an edge-coloured or a layered graph [8], where each layer contains the same set of ‘replica nodes’. Interdependent networks are instead composed of two or more interconnected networks, where each node of a layer is dependent on one or more nodes belonging to the other layers [4]. Several studies have focused on the analysis of structural aspects of multiplex networks [9,10,11]. In particular, the analysis of the overlap between layers of a multiplex can provide valuable information in order to better understand the phenomenology of dynamical processes that occur on top of these systems [12,13] or possible failure cascades [4]. Moreover, the presence of dependencies between layers crucially affects the systemic risk associated with these networks, for instance, in the case of financial or economic systems [14,15].

It must be pointed out that, even in the case of monoplex (i.e., single-layer) networks, the characterization of the aforementioned dynamical processes requires knowledge of the full graph structure. In general, however, issues of confidentiality or limitedness of the data may reduce the knowledge about the network to partial information about nodes. For instance, we may not know the entire set of connections and/or the associated weights, but we may still know the *degree* (number of links) and/or the *strength* (total weight of such links) of each node. Various network reconstruction methods have therefore been developed in order to successfully infer the full topological structure of graphs starting from incomplete information [16,17,18,19,20,21,22,23,24,25,26,27,28,29,30,31]. Unfortunately, most of the current methodologies are applicable only to single-layer networks, leaving an important gap open in the study of multiplex networks. If these techniques were applied to each layer of a multiplex separately, they would by construction fail in replicating the empirical coupling between layers.

One of the most notable attempts of multiplex link prediction has exploited the assumption of so-called hidden geometric correlations [32,33]. According to this approach, multiplex networks can be embedded into a hyperbolic space, such that the coordinates of the nodes represent their so-called popularity (number of connections) and similarity. It has been shown that these coordinates are positively correlated across layers, and the resulting geometric correlations can be exploited to predict (i.e., reconstruct) trans-layer connections. Another related approach to our problem of interest is link prediction [16,34]. While originally designed to infer future connections based on past ones in time-varying networks, link prediction has extended to other types of systems, including multiplex networks. Several techniques have been employed to forecast the links of a layer based on the information resulting from other layers, for instance, in online social networks [35] or co-authorship systems [36]. Similarly to these approaches, our main goal here is that of developing a satisfactory methodology for the reconstruction of multiplex networks from partial topological information, but in a general case when, even for the observable layer, one may not have full information available. This implies that our starting point for probabilistically reconstructing one layer of a multiplex network, given the information about a second layer, will not necessarily be the completely known adjacency matrix for the second layer but in general will only be partial information about the second layer.

Our approach is guided by the following consideration: Clearly, a single-layer network can be seen as a particularly simple case of a multiplex, corresponding to the extreme limit when the number of layers is one. Then, from an entirely general point of view, a method to reconstruct multiplex networks may fail as a result of (a combination of) two factors. On one hand, the method may be unsuccessful because the properties of (some of) the layers are incorrectly reconstructed. This may be due to the method failing on individual layers separately, a circumstance that highlights an intrinsic unreliability of the reconstruction model itself, even when applied at the single-layer limit. On the other hand, the method may succeed in replicating the marginal properties of each layer separately, while it may fail in replicating the interdependencies among layers. In the former case, we do not learn anything useful about whether and how the method can be improved when formulated in the multilayer case. By contrast, the latter situation is quite informative, as it indicates that, if the reconstruction model could be generalized in such a way that its marginal single-layer properties are maintained while at the same time its inter-layer ones are made nontrivial, then it would become a candidate method for reconstructing multiplexes with coupled layers.

Following the above reasoning, we put ourselves in the latter situation and assume that the empirical multiplex is taken from a class of multiplex networks for which a satisfactory ‘marginal’ method capable of reliably reconstructing each layer separately already exists. Then, we investigate whether and how a generalized multiplex method featuring the same marginal properties as the existing single-layer method can be constructed. Building on the recent literature on single-layer network reconstruction methods [18,19,20,21,22,23,24,25,26,27,28,29,30,31], we select the world trade multiplex (WTM) [37] as an ideal example of a multiplex network for our analysis. In the WTM, nodes are countries of the world and links represent trade relationships, disaggregated into different commodities. Each commodity gives rise to a separate layer. The links in each layer are in principle directed (from the exporter to the importer) and weighted (by the dollar value of the trade relationship), even though they are often projected onto undirected and/or unweighted ones. The empirical properties of the WTM have been studied extensively [37,38,39,40,41,42]. If all of the commodities are aggregated together, one obtains a single-layer projection documenting the total trade fluxes among countries [38,39,43,44]. At the disaggregate level, the system exhibits strong inter-layer correlations both in its undirected and directed representations [37,40,41,42,45], thus representing a clear example of correlated multiplexes. In the representation considered here, we use data from refs. [46,47] reporting N=207 countries trading M=96 different commodity classes, each representing a given layer of the multiplex.

The WTM fulfils our criterion stated above, because it has been shown that each of its layers are very closely replicated by a model that takes only local node information as input. Indeed, the purely binary structure of each layer of the WTM can be replicated starting from the knowledge of the degree of each node in that layer (Binary Configuration Model) [48,49], while the weighted structure can be successfully replicated from the knowledge of both the strength and the degree of each node in that layer (Enhanced Configuration Model) [20,40,49]. Other network reconstruction models, based only on the knowledge of node strengths and overall network density [21,22,23,24], have also been successfully applied to the aggregate (monoplex) version of the network of international trade. For the same aggregate version of the network, another class of successful models takes inspiration from the economic literature and is based on the Gross Domestic Product of countries and, optionally, on the geographic inter-country distances [44,50,51]. A very recent subclass of such models constructs link probabilities based on the requirement of invariance under node aggregation [28,29,30,31]. Despite all of these successful approaches to the modelling of either individual layers of the WTM or its single-layer aggregate version, it has been shown that knowledge of the strength and/or degree of each node in each layer is not enough to replicate the coupling between layers [41,42,45], illustrating that even if a marginal reconstruction method is successful in each and every layer separately, it necessarily fails in replicating the multiplex as a whole.

Our strategy in this paper is that of devising a way to preserve the good marginal properties of single-layer reconstruction methods, while at the same time introducing a minimal but effective coupling such that, additionally, various robust inter-layer properties of the multiplex are also replicated. The structure of this paper is as follows: In Section 2 we introduce some preliminary concepts that constrain the range of possible multiplex reconstruction models. In Section 3 we focus on the case of binary multiplexes (both undirected and directed) and develop a multiplex reconstruction method in that case. In Section 4 we move on to weighted multiplexes (again, both undirected and directed) and develop the weighted counterpart of the binary reconstruction method. Finally, in Section 5 we make some concluding remarks.

## 2. Preliminaries

This section establishes some useful criteria which constrain the features of the multiplex reconstruction model we are aiming for.

### 2.1. Beyond Inter-Layer Degree Correlations

To reliably reconstruct a multiplex, we need to identify useful target properties that accurately capture the inter-layer coupling. Various notions of such coupling have been developed in the literature, for instance, in terms of the *correlation of layer activity*, *overlapping degree*, *multiplex assortativity* and *inter-layer degree correlation* [9,10,52,53]. These definitions generalize a situation typically encountered in single-layer networks, where degree correlation is usually computed by looking at the average degree of the first neighbours of a node that have a certain degree (*average nearest neighbour degree*). In the same spirit, notions of multiplex assortativity or inter-layer degree correlation have been developed [9,52,53]. Along the same lines, the inter-layer degree correlation function has been defined as:

(1) $${\bar{k}}^{\alpha }\;\left(k^{\beta }\right)=\sum_{k^{\alpha }}k^{\alpha }P\left(k^{\alpha }|k^{\beta }\right),$$

where $P\left(k^{\alpha }|k^{\beta }\right)$ is the probability that a node with a given degree $k^{\beta }$ in layer β has degree $k^{\alpha }$ in layer α.

It is important to remark that, unfortunately, the above quantities are completely uninformative about the component of inter-layer coupling that goes beyond the mere mutual correlation of the degrees of nodes across different layers [41]. For instance, if the same node is a hub in multiple layers (a property that gives rise to positive inter-layer assortativity), it will automatically produce a significant overlap of links across these layers, even if links in different layers are drawn completely independently. Such an overlap should therefore not be taken as a genuine indication of statistical dependency across layers. This spurious effect, which should be filtered out in order to objectively assess inter-layer dependencies, increases with increasing intra-layer density and increasing heterogeneity of local node properties (i.e., degrees and strengths).

In order to detect ‘true’ inter-layer dependencies that are not merely explained by chance, density, or the local properties of individual nodes, one can construct maximum-entropy null models of multiplexes with independent layers and given node properties [48,50,54]. In these null models, in each layer every node has the same degree (for binary networks) or strength (for weighted networks) that it has in the real multiplex [41]. Apart from these constraints, the maximum-entropy multiplex ensemble is completely random and no dependency is introduced among layers. The expectation values of the multiplexity over the null ensemble can be calculated exactly and used to filter out the undesired effects from the measured values. In previous studies, we have used such null models to define new ‘filtered’ metrics that quantify the intensity of coupling among layers of an undirected multiplex network, introducing the concept of *multiplexity* [41]. This approach can also be extended to multi-layer networks with directed edges [42]. In the directed case, the inter-layer link overlap can manifest itself in terms of both the ‘alignment’ (a phenomenon that we called *multiplexity* in analogy with the undirected case [41]) and the ‘anti-alignment’ (a phenomenon that we called *multireciprocity* as a generalization of the ordinary reciprocity for single-layer networks [55,56]) of links across layers. Since in each layer, links are allowed in both directions between any two nodes, the alignment and the anti-alignment of links across layers do not conflict with each other and can actually coexist.

The above approach has been applied to real-world systems such as the WTM [37,40,41] and the European Airport Multiplex [5]. The results of this analysis indicate that, despite much of the empirically observed multiplexity and multireciprocity being a direct consequence of the inter-layer degree (or strength) correlations, statistically significant inter-layer dependencies remain, which are not captured by any previous metric of inter-layer coupling.

### 2.2. Imposing Dyadic Independence in the Multiplex Model

Our aim is that of introducing a minimal but realistic multiplex model that can reproduce the observed inter-layer dependencies discussed above. Unlike the aforementioned null models, the multiplex model should be characterized by the nontrivial joint probabilities of connection involving multiple layers. We want to develop one such model for binary multiplexes and one for weighted multiplexes in both the undirected and directed cases.

To keep the model as simple as possible, we assume *dyadic independence*: the presence (and weight) of a link connecting a pair of nodes in a given layer does not depend on the presence (and weight) of a link connecting a *different* pair of nodes in the same or in any other layer, although it does depend on the presence (and weight) of the links connecting the same pair of nodes in other layers. If we introduce the term *multidyad* to denote a single pair of nodes ‘replicated’ over all layers of the multiplex (i.e., the set of all single-layer dyads involving the same two nodes), the above assumption might be referred to as *multidyadic independence*. We have already proposed a maximum-entropy model with multidyadic independence [45], moving beyond the aforementioned null models with independent layers and introducing all-to-all couplings among layers, resulting in nontrivial joint probabilities between links in all layers. Here, we are interested in empirically characterizing the pairwise joint probabilities (representing the coupling between two layers) consistent with the full (all-to-all) joint probabilities, such that information about one layer can be retrieved from the information available about another layer, marginalizing over all other layers.

Note that, in directed and binary single-layer networks, a dyad formed by two nodes *i* and *j* can have four different topologies (a single link from *i* to *j*, a single link from *j* to *i*, two reciprocal links between *i* and *j*, or no link at all). This implies that, in a directed binary multiplex with *M* layers, a multidyad can have $4^{M}$ possible topologies. In a directed and weighted single-layer network, even assuming that the weights are non-negative integer numbers (as often done in previous approaches), a dyad can already have an infinity of possible weight-dependent configurations. Correspondingly, a multidyad in a multiplex with *M* layers would have an infinite number of configurations. Similar considerations hold for the undirected case, with the only difference being that a dyad in a single-layer unweighted graph can now have two possible distinct values (a link between *i* and *j* or no link at all).

The assumption of multidyadic independence only restricts the topological properties that individual layers can have, but does not restrict the range of possible dependencies among layers of the multiplex. Moreover, many single-layer networks have in fact been shown to have a structure consistent with dyadic independence [38,39,40,48]. This property is also confirmed by the success of network reconstruction techniques that assume dyadic independence [18,19,25,26,57,58]. Important examples are given precisely by the WTM and other economic networks, whose single-layer structure is largely consistent with dyadic independence [21,22,23,24,27,28,29,30,31,38,40]. Some social [59] and biological [60,61] networks have also been found to exhibit a similar property. It should be noted that dyadic independence is also present in more complicated models with a core–periphery or community structure, such as the (degree-corrected [62]) Stochastic Block Model [63]. It may however be lost in models with higher-order interactions [64]. Some key results obtained for single-layer networks consistent with dyadic independence are preliminarily summarized in the next subsection and are the starting point for the definition of our multiplex model in Section 3.

### 2.3. Choice of the Single-Layer Marginal Properties of the Reconstruction Method

Let us consider binary multiplexes for the moment. The marginal—i.e., unconditional on the presence of any other link in any other layer—probability that a (possibly directed) link from node *i* to node *j* exists in layer α is

(2) $$p_{ij}^{\alpha }\equiv P\left(a_{ij}^{\alpha }=1\right)=\langle a_{ij}^{\alpha }\rangle .$$

Due to our assumption of multidyadic independence, the information that is integrated away in obtaining the marginal probability $p_{ij}^{\alpha }$ does not involve other pairs of nodes (joint probabilities involving multiple pairs of nodes would in any case factorize into products of marginal probabilities of invididual pairs of nodes), but it does involve other layers. In other words, $p_{ij}^{\alpha }$ does not contain information about the inter-layer dependencies that we want to model. This marginal model is not actually an essential ingredient of our multiplex model and can be in some sense ‘outsourced’. It can therefore be chosen to be specified by any convenient single-layer network model that satisfactorily reproduces a set of desired marginal topological properties of layer α. The only basic property we require from $p_{ij}^{\alpha }$ is that the degree sequence is among such desired properties. In other words, if

(3) $$\begin{array}{c} k_{i}^{\alpha }=\sum_{j\neq i}a_{ij}^{\alpha } \end{array}$$

denotes the empirical single-layer degree, we require that, for each node *i* and each layer α, the expected degree $\langle k_{i}^{\alpha }\rangle =\sum_{j\neq i}p_{ij}^{\alpha }$ closely matches the empirical degree $k_{i}^{\alpha }$:

(4) $$\langle k_{i}^{\alpha }\rangle =\sum_{j\neq i}p_{ij}^{\alpha }\approx k_{i}^{\alpha }\;\forall i.$$

For instance, if the Binary Configuration Model [48,49] is chosen as the marginal single-layer reconstruction method for each layer α, the above criterion is strictly verified, since that model assumes that the degree of each node is known and that the $p_{ij}^{\alpha }$ can be *constructed* as the maximum-entropy probability such that

(5) $$\langle k_{i}^{\alpha }\rangle =\sum_{j\neq i}p_{ij}^{\alpha }=k_{i}^{\alpha }\;\forall i.$$

Another recent example where the above equality is exactly realized arises in the context of network renormalization and makes use of a probability that is not maximum-entropy, but invariant under node aggregation [31]. Other marginal reconstruction methods, which relax the hypothesis that the degree of each node is known, use other node-specific pieces of information, plus some proxy of the overall network density, to construct a $p_{ij}^{\beta }$ such that Equation (4) is in any case realized [21,22,23,24,25,26,27,28,29,30,44,51]. The above examples have all been shown to provide reliably reconstructed networks [21,22,23,24,27,28,29,30,31].

## 3. Binary Multiplex Model

In this dection, we develop our analytical framework and introduce the multiplex reconstruction method in the case where the layers are binary graphs. We also show the application of the method to the binarized version of the WTM [41,47].

The requirement that layers are dependent implies that joint connection probabilities involving the same pairs of nodes but different layers should *not* trivially factorize into products of marginal probabilities. We therefore need to introduce generic joint probabilities that involve multiple layers. In general, even if we are assuming multidyadic independence, for each pair of nodes we should consider the joint probabilities of all combinations of links across all layers together, i.e., (in the jargon of multiplex networks [54]) the probabilities of all possible *multilinks* involving the same two nodes—as in our model with all-to-all couplings between layers [45]. However, as we mentioned, in multiplexes with directed links, a multidyad can have $4^{M}$ possible topologies, i.e., $4^{M}$ possible multilinks. For each pair of nodes, fully specifying the joint connection probabilities across all layers would require the specification of a different probability for each of these multilinks, with the only constraint being that the $4^{M}$ probabilities sum up to one. This would lead to the definition of $4^{M}-1$ probabilities. While this operation is feasible and insightful in the most studied case of a multiplex with two layers only, it becomes increasingly challenging (and decreasingly transparent) as *M* increases.

By contrast, we want to keep our approach feasible and useful (both from a modelling and from a network reconstruction perspective) even in the case of a very large number of layers, for which our formalism based on multiplexity and multireciprocity matrices fully shows its advantages. Therefore we take the following parsimonious approach: For a given pair of nodes, we start from the definition of two joint (and conditional) probabilities that fully characterize both the multiplexity and the reciprocity properties of a single pair of layers, and then consider the set of such probabilities for all the $M^{2}$ pairs of layers (including a layer with itself) of the multiplex. This leads to a set of only $2M^{2}$ probabilities defining the directed multiplex model (but still for a single pair of nodes). This set represents the relevant projection (or marginalization) of the full set of $4^{M}-1$ multilink probabilities. The quadratic (as opposed to exponential) growth of the number of probabilities with the number of layers makes our approach appealing and manageable. Moreover, we will show that, at least in the empirical case study considered here, the conditional probabilities are approximately independent of the particular pair of nodes, making the information contained in the multiplexity and multireciprocity matrices sufficient in order to fully characterize the dependencies among the layers. Remarkably, this also means that the number of relevant probabilities remains $2M^{2}$ (equal to the total number of entries in the multiplexity and multireciprocity matrices) independently of the number *N* of nodes in the multiplex. Similar considerations can be made for the undirected systems.

We recall that, as reported in [41], in the undirected binary case, the multiplexity reads:

(6) $$m_{b}^{\alpha \beta }=\frac{2\sum_{i}\sum_{j<i}\min\left\{a_{ij}^{\alpha },a_{ij}^{\beta }\right\}}{L^{\alpha }+L^{\beta }}=\frac{2\sum_{i}\sum_{j<i}a_{ij}^{\alpha }a_{ij}^{\beta }}{L^{\alpha }+L^{\beta }}=\frac{2L^{\alpha ⇉\beta }}{L^{\alpha }+L^{\beta }},$$

where $a_{ij}^{\alpha }$ denotes the entry of the adjacency matrix of layer α, $L^{\alpha }=\sum_{i}\sum_{j<i}a_{ij}^{\alpha }$ is the number of links in that layer and $L^{\alpha ⇉\beta }=\sum_{i}\sum_{j<i}a_{ij}^{\alpha }a_{ij}^{\beta }$ counts the number of links present in both layers α and β between the same pairs of nodes. This notation is somewhat redundant at this stage, but on the other hand it allows for an easier generalization to the directed case, as we will show later. So, $m_{b}^{\alpha \beta }$ ranges between 0 and 1 and represents a normalized overlap between pairs of layers of a multiplex. As mentioned in the Introduction, in the directed case we must take into account both the ‘aligned’ and the ‘anti-aligned’ overlap. Hence, in [42], we defined the binary directed multiplexity and multireciprocity as

(7) $$m_{b}^{\alpha \beta }=\frac{2\sum_{i}\sum_{j\neq i}\min\left\{a_{ij}^{\alpha },a_{ij}^{\beta }\right\}}{L^{\alpha }+L^{\beta }}=\frac{2\sum_{i}\sum_{j\neq i}a_{ij}^{\alpha }a_{ij}^{\beta }}{L^{\alpha }+L^{\beta }}=\frac{2L^{\alpha ⇉\beta }}{L^{\alpha }+L^{\beta }}$$

and

(8) $$r_{b}^{\alpha \beta }=\frac{2\sum_{i}\sum_{j\neq i}\min\left\{a_{ij}^{\alpha },a_{ji}^{\beta }\right\}}{L^{\alpha }+L^{\beta }}=\frac{2\sum_{i}\sum_{j\neq i}a_{ij}^{\alpha }a_{ji}^{\beta }}{L^{\alpha }+L^{\beta }}=\frac{2L^{\alpha ⇆\beta }}{L^{\alpha }+L^{\beta }}$$

respectively, where $L^{\alpha }=\sum_{i}\sum_{j\neq i}a_{ij}^{\alpha }$ is the total number of directed links in layer α, $L^{\alpha ⇉\beta }=\sum_{i}\sum_{j\neq i}a_{ij}^{\alpha }a_{ij}^{\beta }$ is the number of directed links present in both layers α and β between the same pairs of nodes, while $L^{\alpha ⇆\beta }=\sum_{i}\sum_{j\neq i}a_{ij}^{\alpha }a_{ji}^{\beta }$ is the number of directed links present in α which are reciprocated in β over all the possible pairs of vertices.

In the previous sections we stressed the importance of the inter-layer link coupling for the characterization of a real-world multiplex. We now pave the way for realistic (undirected and directed) binary models that can capture the observed features in the particular case of the WTM. Once more, one should not confuse these realistic models with the null models used in other contexts [65].

### 3.1. Undirected Binary Model

We start with the definition of one of the new metrics that will allow us to properly describe the inter-layer coupling of a multiplex. We define the empirical *multiplexed degree* as

(9) $$k_{i}^{\alpha ⇉\beta }=\sum_{j\neq i}a_{ij}^{\alpha }a_{ij}^{\beta }.$$

If we look at Equation (6), we immediately see that, as compared to the global quantity $m_{b}^{\alpha ,\beta }$, the multiplexed degree $k_{i}^{\alpha ⇉\beta }$ provides an even more detailed, local quantification of the multiplexity.

**Figure 1:**
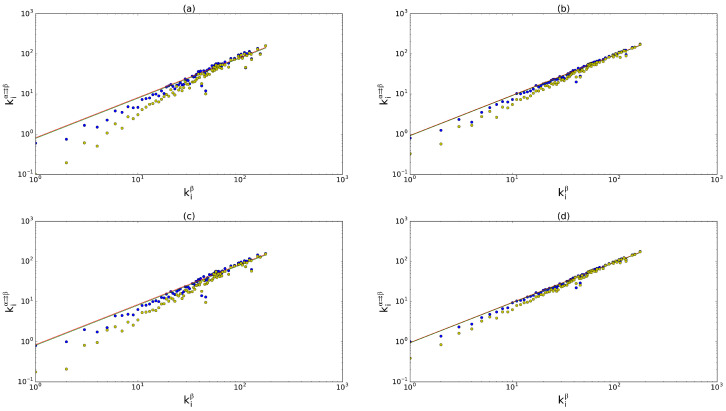
Degree $k_{i}^{\beta }$ (where β is the layer of trade in cereals) versus inter-layer multiplexed degree $k_{i}^{\alpha ⇉\beta }$ against four other layers α: inorganic chemicals (**a**), plastics (**b**), iron and steel (**c**), and electric machinery (**d**). Blue dots: real data, which approximately follow the empirical linear relation in Equation (10); yellow dots: expected multiplexed degree according to the uncorrelated model; lower green line: expected trend according to the theoretical slope $u^{\alpha \beta }$ in Equation (24); upper red line (when discernible): best fit. In all the cases, $R^{2}>0.93$ for both of the curves. It should be noted that we fit the empirical data with lines of the form y=a·x, and only after we plot the results in log–log scale.

In what follows, we first establish an empirically robust pattern displayed by $k_{i}^{\alpha ⇉\beta }$ and then select it as one of the target properties that a multiplex reconstruction model should replicate, in addition to the desired marginal single-layer network properties. Figure 1 reports the scatter plot of $k_{i}^{\alpha ⇉\beta }$ versus $k_{i}^{\beta }$ for four representative pairs of commodities. We clearly see an approximate linear trend of the type

(10) $$k_{i}^{\alpha ⇉\beta }\approx u^{\alpha \beta }k_{i}^{\beta }.$$

We observed similar plots for the other pairs of layers as well. The robustness of this pattern motivates us to look for a multiplex model able to replicate it.

We define the joint probability $p_{ij}^{\alpha ⇉\beta }$ for the simultaneous presence of a link from node *i* to node *j* in layer α and of a corresponding link in layer β:

(11) $$p_{ij}^{\alpha ⇉\beta }\equiv P\left(a_{ij}^{\alpha }=1\cap a_{ij}^{\beta }=1\right)=\langle a_{ij}^{\alpha }a_{ij}^{\beta }\rangle =p_{ij}^{\beta ⇉\alpha }.$$

Using $p_{ij}^{\alpha ⇉\beta }$ and the aforementioned $p_{ij}^{\beta }$, we can also obtain the *conditional* probability $u_{ij}^{\alpha \beta }$ that a link from *i* to *j* exists in layer α, given that the corresponding link exists in layer β:

(12) $$u_{ij}^{\alpha \beta }\equiv P\left(a_{ij}^{\alpha }=1|a_{ij}^{\beta }=1\right)=p_{ij}^{\alpha ⇉\beta }/p_{ij}^{\beta }.$$

We call $u_{ij}^{\alpha \beta }$ the *multiplexity probability*. Note that, while $p_{ij}^{\alpha ⇉\beta }$ is symmetric under the exchange of α and β, $u_{ij}^{\alpha \beta }$ is not; indeed, we have

(13) $$p_{ij}^{\alpha ⇉\beta }=u_{ij}^{\alpha \beta }p_{ij}^{\beta }=u_{ij}^{\beta \alpha }p_{ij}^{\alpha }=p_{ij}^{\beta ⇉\alpha }.$$

Furthermore $p_{ij}^{\alpha ⇉\beta }$ depends, at least in the general case, both on the pair of nodes and on the pair of layers. Given the previous definitions, the expected value of the multiplexed degree becomes

(14) $$\begin{array}{c} \langle k_{i}^{\alpha ⇉\beta }\rangle =\sum_{j\neq i}\langle a_{ij}^{\alpha }a_{ij}^{\beta }\rangle =\sum_{j\neq i}p_{ij}^{\alpha ⇉\beta }=\sum_{j\neq i}u_{ij}^{\alpha \beta }p_{ij}^{\beta }=\sum_{j\neq i}u_{ij}^{\beta \alpha }p_{ij}^{\alpha }. \end{array}$$

The main goal consists of understanding the structure of $u_{ij}^{\alpha \beta }$, which is the crucial quantity responsible for the coupling among layers. The presence of a nontrivial $u_{ij}^{\alpha \beta }$ in the present multiplex model implies that any $p_{ij}^{\beta }$ coming from a single-layer model (see previous section) should be interpreted as a marginal probability resulting from a more realistic model where the presence of links across all layers is governed by a joint distribution for the entire multiplex. In other words, $u_{ij}^{\alpha \beta }$ allows us to extend any desired single-layer model to a truly multiplex model with nontrivial coupling among layers. The trivial case of independent layers can be easily recovered by setting

(15) $${\left[u_{ij}^{\alpha \beta }\right]}_{unc}=p_{ij}^{\alpha },$$

since here the presence of the link in layer β does not affect the connection probability in layer α. In such a case, the expected multiplexed degree becomes

(16) $${\langle k_{i}^{\alpha ⇉\beta }\rangle }_{unc}=\sum_{j\neq i}p_{ij}^{\alpha }p_{ij}^{\beta }.$$

From Equation (6). it should be noted that, if such an uncoupled model were used to generate the multiplex, the expected value of the multiplexity $m_{b}^{\alpha \beta }$ would be zero. Yet, if Equation (4) holds, then the inter-layer degree correlation function defined in Equation (1) would be replicated. This shows that such a correlation function is not informative about the genuine inter-layer dependencies which go beyond the degree–degree correlations across the layers of the multiplex. By contrast, the multiplexity $m_{b}^{\alpha \beta }$ is informative, confirming the argument that led us to its introduction in ref. [41].

To build a minimal model that can reproduce the observed level of similarity (i.e., multiplexity) between layers of the multiplex, we require that the robust empirical trend encapsulated in Equation (10) is replicated. Looking at Equations (4) and (14), and imposing Equation (10), this requirement implies that the conditional probability $u_{ij}^{\alpha \beta }$ should be approximately independent of the pair of nodes:

(17) $$\begin{array}{c} u_{ij}^{\alpha \beta }=\frac{p_{ij}^{\alpha ⇉\beta }}{p_{ij}^{\beta }}=\frac{\langle a_{ij}^{\alpha }a_{ij}^{\beta }\rangle }{\langle a_{ij}^{\beta }\rangle }\approx u^{\alpha \beta }. \end{array}$$

Since the transformation i↦j together with α↦β keeps the quantities unaffected, we also have

(18) $$\begin{array}{c} u^{\alpha \beta }\langle a_{ij}^{\beta }\rangle \approx \langle a_{ij}^{\alpha }a_{ij}^{\beta }\rangle =\langle a_{ij}^{\beta }a_{ij}^{\alpha }\rangle \approx q^{\beta \alpha }\langle a_{ij}^{\alpha }\rangle . \end{array}$$

Summing over *i* and *j*, we get

(19) $$\begin{array}{c} u^{\alpha \beta }L^{\beta }\approx u^{\beta \alpha }L^{\alpha }. \end{array}$$

and from (17) we immediately have

(20) $$\begin{array}{c} u^{\alpha \beta }\langle a_{ij}^{\beta }\rangle \approx \langle a_{ij}^{\alpha }a_{ij}^{\beta }\rangle . \end{array}$$

Summing over *i* and *j* and inverting, we obtain

(21) $$\begin{array}{c} u^{\alpha \beta }\approx \frac{\sum_{i}\sum_{j<i}\langle a_{ij}^{\alpha }a_{ij}^{\beta }\rangle }{\sum_{i}\sum_{j<i}\langle a_{ij}^{\beta }\rangle }=\frac{\sum_{i}\sum_{j<i}a_{ij}^{\alpha }a_{ij}^{\beta }}{L^{\beta }}. \end{array}$$

The above relations allow us to express twice the inverse of (6) as

(22) $$\begin{array}{c} \frac{2}{m_{b}^{\alpha \beta }}=\frac{L^{\alpha }+L^{\beta }}{\sum_{i}\sum_{j<i}a_{ij}^{\alpha }a_{ij}^{\beta }}\approx \frac{1}{u^{\alpha \beta }}+\frac{1}{u^{\beta \alpha }}, \end{array}$$

where $m_{b}^{\alpha \beta }$ is measured from the multiplex data while $u^{\alpha \beta }$ is derived from the slope of the empirical linear relationship between $k_{i}^{\beta }$ and $k_{i}^{\alpha ⇉\beta }$. Thus, we find that $m_{b}^{\alpha \beta }$ is approximately the harmonic mean of the conditional probabilities $u^{\alpha \beta }$ and $u^{\beta \alpha }$. Applying Equation (19) to the previous expression, we get

(23) $$\begin{array}{ccc} \frac{2}{m_{b}^{\alpha \beta }} & \approx & \frac{1}{u^{\alpha \beta }}\left(1+\frac{u^{\alpha \beta }}{u^{\beta \alpha }}\right)\\ & \approx & \frac{1}{u^{\alpha \beta }}\left(1+\frac{L^{\alpha }}{L^{\beta }}\right)\\ & = & \frac{L^{\alpha }+L^{\beta }}{u^{\alpha \beta }L^{\beta }}. \end{array}$$

Hence, the value of the slope in the plots of $k_{i}^{\alpha ⇉\beta }$ vs. $k_{i}^{\beta }$ is predicted to be

(24) $$\begin{array}{c} u^{\alpha \beta }\approx \frac{L^{\alpha }+L^{\beta }}{2L^{\beta }}m_{b}^{\alpha \beta }. \end{array}$$

Indeed, in Figure 1 we show that the best-fit curves almost coincide with the expected ones with a slope calculated independently from Equation (24). Furthermore, we also show that the model assuming independent layers, as in Equations (15) and (16), produces values of the multiplexed degree that are systematically lower than the empirical ones.

From the previous analysis, it turns out phenomenologically that the minimal model one can design in order to reproduce the (local) observed values of the multiplexed degree requires only the (global) information about the total number of multiplexed links $L^{\alpha ⇉\beta }$ for any ordered pair of layers (α,β) (together with the aforementioned degree sequences in each layer). In other words, a reliable network reconstruction method for the class of multiplexes we are focusing on here requires as input information a reconstruction model that works successfully on each layer separately, plus the M(M−1)/2 values of $L^{\alpha ⇉\beta }$, for all pairs of layers. These values are the numerators of the entries of the so-called (binary) *multiplexity matrix* [41]. If the reconstruction model is chosen to be the Binary Configuration Model [48,49] or the recent model in [31], then the overall input information reduces to the degree sequence ${\vec{k}}^{\alpha }$ for each layer α, plus the values $L^{\alpha ⇉\beta }$ for each pair of layers.

### 3.2. Directed Binary Model

As said in the Introduction, in the directed case we should take into account that the inter-layer coupling can intervene both in terms of alignment and anti-alignment. Hence, we not only have to extend the notion of *multiplexed degree* to the directed case, but also to introduce the quantity dubbed the *multireciprocated degree*. It is indeed straightforward to exploit the same approach to analyze the patterns of multiplexity and multireciprocity in the directed case. The main difference w.r.t. the undirected case will consist of the definition of two separate conditional probabilities. We start by defining the quantities that we will measure on the real multiplex network, namely the in-degree

(25) $$\begin{array}{c} k_{i}^{\alpha ,in}=\sum_{j\neq i}a_{ji}^{\alpha } \end{array}$$

and the out-degree

(26) $$\begin{array}{c} k_{i}^{\alpha ,out}=\sum_{j\neq i}a_{ij}^{\alpha }. \end{array}$$

In analogy with the undirected model, we assume we can start from a marginal single-layer model characterized by the probability $p_{ij}^{\beta }=\langle a_{ij}^{\beta }\rangle$ and that a *directed* link from node *i* to node *j* exists. The only thing we require from $p_{ij}^{\beta }$ is that it reliably replicates the in- and out-degree of each node *i* in layer β:

(27) $$\begin{array}{ccc} \langle k_{i}^{\alpha ,in}\rangle & = & \sum_{j\neq i}p_{ji}^{\alpha }\approx k_{i}^{\alpha ,in}\;\forall i, \end{array}$$

(28) $$\begin{array}{ccc} \langle k_{i}^{\alpha ,out}\rangle & = & \sum_{j\neq i}p_{ij}^{\alpha }\approx k_{i}^{\alpha ,out}\;\forall i, \end{array}$$

generalizing the corresponding criterion in Equation (4).

We also define the multiplex quantities that extend the ones introduced in the undirected case, i.e., the *multiplexed degree*

(29) $$k_{i}^{\alpha ⇉\beta }=\sum_{j\neq i}a_{ij}^{\alpha }a_{ij}^{\beta }$$

and the *multireciprocated degree*

(30) $$k_{i}^{\alpha ⇄\beta }=\sum_{j\neq i}a_{ij}^{\alpha }a_{ji}^{\beta }.$$

**Figure 2:**
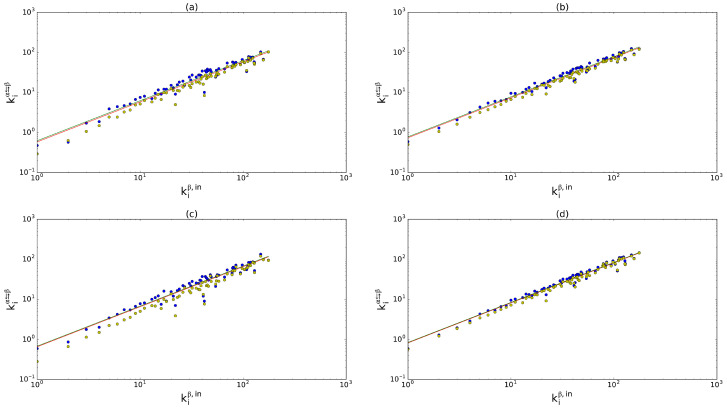
In-degree $k_{i}^{\beta ,in}$ (where β is the layer of trade in cereals) versus inter-layer multireciprocated degree $k_{i}^{\alpha ⇄\beta }$ against four other layers α: inorganic chemicals (**a**), plastics (**b**), iron and steel (**c**), and electric machinery (**d**). Blue dots: real data, which approximately follow the empirical linear relation in Equation (32); yellow dots: expected multireciprocated degree according to the uncorrelated model; lower green line: expected trend according to the theoretical slope $v^{\alpha \beta }$ in Equation (40); upper red line (when discernible): best fit. In all of the cases, $R^{2}>0.95$ for both the curves. It should be noted that we fit the empirical data with lines of the form y=a·x and only after we plot the results in log–log scale.

It is possible to generalize the argument explained in the previous subsection. Also in this case, we find that $k_{i}^{\alpha ⇉\beta }$ and $k_{i}^{\alpha ⇄\beta }$ are in almost linear relation with, respectively, $k_{i}^{\beta ,out}$ (not shown, as it is very similar to the undirected case) and $k_{i}^{\beta ,in}$ (Figure 2). Therefore we can set

(31) $$k_{i}^{\alpha ⇉\beta }\approx u^{\alpha \beta }k_{i}^{\beta ,out}$$

and

(32) $$k_{i}^{\alpha ⇄\beta }\approx v^{\alpha \beta }k_{i}^{\beta ,in}.$$

The presence of two different multiplex quantities leads to the definition of two distinct joint probabilities. Specifically,

(33) $$p_{ij}^{\alpha ⇉\beta }\equiv P\left(a_{ij}^{\alpha }=1\cap a_{ij}^{\beta }=1\right)=\langle a_{ij}^{\alpha }a_{ij}^{\beta }\rangle =p_{ij}^{\beta ⇉\alpha }$$

is the probability for the simultaneous presence of a link from node *i* to node *j* in layer α and of a corresponding link (with the same direction) in layer β, while

(34) $$p_{ij}^{\alpha ⇄\beta }\equiv P\left(a_{ij}^{\alpha }=1\cap a_{ji}^{\beta }=1\right)=\langle a_{ij}^{\alpha }a_{ji}^{\beta }\rangle =p_{ji}^{\beta ⇄\alpha }$$

is the probability of having a link from node *i* to node *j* in layer α and a link in the opposite direction in layer β. Consequently, from these joint probabilities and the marginal single-layer probabilities we can derive both the *conditional* probability

(35) $$u_{ij}^{\alpha \beta }\equiv P\left(a_{ij}^{\alpha }=1|a_{ij}^{\beta }=1\right)=p_{ij}^{\alpha ⇉\beta }/p_{ij}^{\beta }$$

that a link from *i* to *j* exists in layer α, given that the corresponding link exists in layer β, and the probability

(36) $$v_{ij}^{\alpha \beta }\equiv P\left(a_{ij}^{\alpha }=1|a_{ji}^{\beta }=1\right)=p_{ij}^{\alpha ⇄\beta }/p_{ji}^{\beta }$$

of having a link from *i* to *j* in α, given that a link from *j* to *i* exists in layer β. We call $u_{ij}^{\alpha \beta }$ the *multiplexity probability* and $v_{ij}^{\alpha \beta }$ the *multireciprocity probability*. These probabilities lead to the separate notions of expected *multiplexed* and *multireciprocated degree*, defined respectively as

(37) $$\begin{array}{c} \langle k_{i}^{\alpha ⇉\beta }\rangle =\sum_{j\neq i}\langle a_{ij}^{\alpha }a_{ij}^{\beta }\rangle =\sum_{j\neq i}p_{ij}^{\alpha ⇉\beta }=\sum_{j\neq i}u_{ij}^{\alpha \beta }p_{ij}^{\beta }=\sum_{j\neq i}u_{ij}^{\beta \alpha }p_{ij}^{\alpha } \end{array}$$

and

(38) $$\begin{array}{c} \langle k_{i}^{\alpha ⇄\beta }\rangle =\sum_{j\neq i}\langle a_{ij}^{\alpha }a_{ji}^{\beta }\rangle =\sum_{j\neq i}p_{ij}^{\alpha ⇄\beta }=\sum_{j\neq i}v_{ij}^{\alpha \beta }p_{ji}^{\beta }=\sum_{j\neq i}v_{ij}^{\beta \alpha }p_{ji}^{\alpha }. \end{array}$$

Analogously to the undirected case, $u_{ij}^{\alpha \beta }$ and $v_{ij}^{\alpha \beta }$ are driving the real coupling among the layers of the system, while the single-layer probabilities $p_{ij}^{\alpha }$ can be freely chosen starting from any network model that correctly reproduces the marginal topology of the considered layer. For instance, we may choose the Directed Binary Configuration Model [48,49], for which Equations (27) and (28) hold with a strict equality sign, or some of its relaxed versions that assume less input information [21,22,23].

With the same reasoning as in the previous subsection, it is possible to show that the value of the slope in the plots of $k_{i}^{\alpha ⇉\beta }$ vs. $k_{i}^{\beta ,out}$ is predicted to be equal to

(39) $$\begin{array}{c} u^{\alpha \beta }\approx \frac{L^{\alpha }+L^{\beta }}{2L^{\beta }}m_{b}^{\alpha \beta }, \end{array}$$

where $m_{b}^{\alpha \beta }$ is the binary directed multiplexity defined in Equation (7), while the slope in the plots of $k_{i}^{\alpha ⇄\beta }$ vs. $k_{i}^{\beta ,in}$ is, according to the model, equal to

(40) $$\begin{array}{c} v^{\alpha \beta }\approx \frac{L^{\alpha }+L^{\beta }}{2L^{\beta }}r_{b}^{\alpha \beta }, \end{array}$$

where $r_{b}^{\alpha \beta }$ is the binary multireciprocity defined in Equation (8). As shown in Figure 2 for the multireciprocated degree (the corresponding plot referring to the multiplexed degree is not reported, as it is very similar to the undirected case), the best-fit curves are well-modelled by the expected ones. We also show the results of the uncorrelated model, again producing values of the multireciprocated degree which are systematically lower than the observed values.

It therefore turns out that an appropriate multiplex reconstruction method for the class of directed multi-layer networks that we are considering is based on the information about the in- and out-degree sequences of each layer combined with the entries of the matrices $L^{\alpha ⇉\beta }$ and $L^{\alpha ⇄\beta }$ for any pair of layers.

## 4. Weighted Multiplex Model

In the case of weighted multiplex networks, the marginal (i.e., single-layer) quantity we will focus on is the weight $w_{ij}^{\alpha }$ associated with any (possibly directed) link between *i* and *j* in layer α, together with its expected value $\langle w_{ij}^{\alpha }\rangle$ under the chosen reconstruction model. At the same time, we can still consider the binary variable $a_{ij}^{\alpha }$, where $a_{ij}^{\alpha }=1$ if $w_{ij}^{\alpha }>0$ and $a_{ij}^{\alpha }=0$ if $w_{ij}^{\alpha }=0$. The expected value of this binary variable is the link probability $p_{ij}^{\alpha }=\langle a_{ij}^{\alpha }\rangle$ representing the chance that nodes *i* and *j* are connected by a link, irrespective of the weight of the latter. We still assume multidyadic independence, i.e., the fact that the existence and weight of the link between nodes *i* and *j* do not depend on the existence and weight of any other link.

We require that the marginal quantities $\langle w_{ij}^{\alpha }\rangle$ are not influenced by the inter-layer coupling that we will add later. They can therefore be set equal to the expectation values generated by any marginal model that can correctly reproduce the structure of layer α. To be successful in reconstructing each layer, such a method should reliably replicate both the topology and the weighted structure of the corresponding network. It has been shown that the Weighted Configuration Model [66], producing random weighted networks where each node has a given strength (but not a given degree), does not achieve this goal, as it gives rise to (almost) complete graphs [20,39]. Instead, a successful model is the Enhanced Configuration Model [40,49], constraining both the degree and the strength sequence of the observed graph. Using the latter model, for every vertex *i* we consider two constraints for each layer α, namely the strength $s_{i}^{\alpha }$ and the degree $k_{i}^{\alpha }$, defined as

(41) $$\begin{array}{ccc} s_{i}^{\alpha } & = & \sum_{j\neq i}w_{ij}^{\alpha }, \end{array}$$

(42) $$\begin{array}{ccc} k_{i}^{\alpha } & = & \sum_{j\neq i}a_{ij}^{\alpha } \end{array}$$

respectively. The Enhanced Configuration Model [40,49] is constructed as the maximum-entropy ensemble of weighted graphs that realize the expected constraints exactly:

(43) $$\begin{array}{ccc} \langle s_{i}^{\alpha }\rangle & = & \sum_{j\neq i}\langle w_{ij}^{\alpha }\rangle =s_{i}^{\alpha }\;\forall i, \end{array}$$

(44) $$\begin{array}{ccc} \langle k_{i}^{\alpha }\rangle & = & \sum_{j\neq i}p_{ij}^{\alpha }=k_{i}^{\alpha }\;\forall i. \end{array}$$

Similarly to the binary case, these constraints can be relaxed in such a way that the required input information is considerably reduced. For instance, the methods proposed in refs. [21,22,23] require much less input information but are still such that

(45) $$\begin{array}{ccc} \langle s_{i}^{\alpha }\rangle & = & \sum_{j\neq i}\langle w_{ij}^{\alpha }\rangle \approx s_{i}^{\alpha }\;\forall i, \end{array}$$

(46) $$\begin{array}{ccc} \langle k_{i}^{\alpha }\rangle & = & \sum_{j\neq i}p_{ij}^{\alpha }\approx k_{i}^{\alpha }\;\forall i, \end{array}$$

and have recently been found to provide the best reconstruction methods for monoplex weighted networks from limited information [25,57,58].

In the weighted case, the assumption of dependency between layers means that the joint probability of observing a given weight $w_{ij}^{\alpha }$ between *i* and *j* in layer α together with a weight $w_{ij}^{\beta }$ in β does not factorize into two separate single-layer probabilities. In previous studies [67] this issue has been tackled by introducing the concept of *multistrength*; however, as already explained for the binary case, this approach is practically feasible only in the case of multiplex networks with a (very) limited number of layers. On the contrary, our multiplex reconstruction technique appears to also be useful when applied to multi-graphs possessing a larger number of layers, as it requires as input the strength sequence of the various layers and the multiplexity/multireciprocity matrices (both growing like $M^{2}$). This quadratic growth in the number of layers (opposed to the exponential growth shown by the multistrength method), combined with the phenomenological observation that the conditional probabilities are again independent of the considered pair of nodes, makes our approach very promising.

As we noted previously, our reconstruction method builds on the notions of *weighted multiplexity and multireciprocity*. In particular, in the undirected case, we can exploit the following definition of weighted multiplexity $m_{w}^{\alpha \beta }$ introduced in [41]:

(47) $$m_{w}^{\alpha \beta }=\frac{2\sum_{i}\sum_{j<i}\min\left\{w_{ij}^{\alpha },w_{ij}^{\beta }\right\}}{W^{\alpha }+W^{\beta }}=\frac{2W^{\alpha ⇉\beta }}{W^{\alpha }+W^{\beta }},$$

where $w_{ij}^{\alpha }$ is the entry of the weighted adjacency matrix of layer α, $W^{\alpha }=\sum_{i}\sum_{j<i}w_{ij}^{\alpha }$ is the total weight associated with the links in that layer, and $W^{\alpha ⇉\beta }=\sum_{i}\sum_{j<i}\min\left\{w_{ij}^{\alpha },w_{ij}^{\beta }\right\}$ represents the ‘shared weight’ between layers α and β. In analogy with the binary case, $m_{w}^{\alpha \beta }$ ranges between 0 and 1 and represents a normalized weighted overlap between pairs of layers of the multi-graph. In the directed case, instead, we have to consider the overlap in both directions. In [42] we defined the weighted directed multiplexity and multireciprocity as

(48) $$m_{w}^{\alpha \beta }=\frac{2\sum_{i}\sum_{j\neq i}\min\left\{w_{ij}^{\alpha },w_{ij}^{\beta }\right\}}{W^{\alpha }+W^{\beta }}=\frac{2W^{\alpha ⇉\beta }}{W^{\alpha }+W^{\beta }}$$

and

(49) $$r_{w}^{\alpha \beta }=\frac{2\sum_{i}\sum_{j\neq i}\min\left\{w_{ij}^{\alpha },w_{ji}^{\beta }\right\}}{W^{\alpha }+W^{\beta }}=\frac{2W^{\alpha ⇄\beta }}{W^{\alpha }+W^{\beta }}$$

respectively, where $W^{\alpha }=\sum_{i}\sum_{j\neq i}w_{ij}^{\alpha }$ is the total weight of the links in layer α, $W^{\alpha ⇉\beta }=\sum_{i}\sum_{j\neq i}\min\left\{w_{ij}^{\alpha },w_{ij}^{\beta }\right\}$ is the ‘shared total weight’ between layers α and β, and $W^{\alpha ⇆\beta }=\sum_{i}\sum_{j\neq i}\min\left\{w_{ij}^{\alpha },w_{ji}^{\beta }\right\}$ is the ‘shared reciprocated weight’ between layers α and β.

In the following sections we will show a method to reconstruct the WTM from single-layer information, exploiting the knowledge of the aforementioned multiplexity and multireciprocity matrices.

### 4.1. Undirected Weighted Model

**Figure 3:**
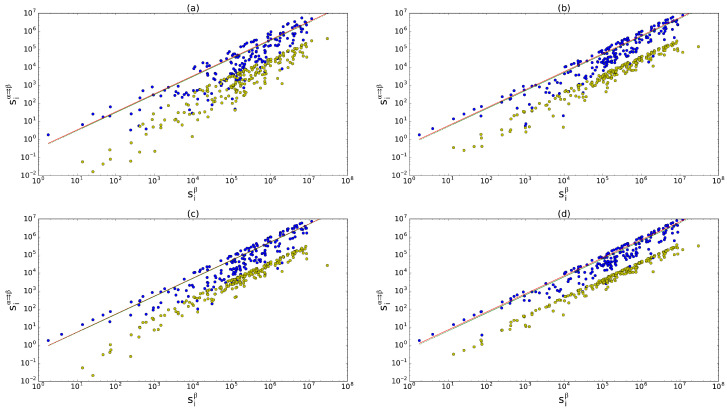
Strength $s_{i}^{\beta }$ (where β is the layer of trade in cereals) versus inter-layer multiplexed strength $s_{i}^{\alpha ⇉\beta }$ against four other layers α: inorganic chemicals (**a**), plastics (**b**), iron and steel (**c**), and electric machinery (**d**). Blue dots: real data, which approximately follow the empirical linear relation in Equation (52); yellow dots: expected multiplexed strength according to the uncorrelated model; lower green line: expected trend according to the theoretical slope $U^{\alpha \beta }$ in Equation (61); upper red line (when discernible): best fit. In all the cases, $R^{2}>0.92$ for both of the curves. It should be noted that we fit the empirical data with lines of the form y=a·x and only after we plot the results in log–log scale.

In this section, we will focus on the relation between the single-layer strength, defined as

(50) $$\begin{array}{c} s_{i}^{\alpha }=\sum_{j\neq i}w_{ij}^{\alpha }, \end{array}$$

and the *multiplexed strength*, defined as

(51) $$\begin{array}{c} s_{i}^{\alpha ⇉\beta }=\sum_{j\neq i}w_{ij}^{\alpha ⇉\beta }\equiv \sum_{j\neq i}\min\left\{w_{ij}^{\alpha },w_{ij}^{\beta }\right\}, \end{array}$$

where $w_{ij}^{\alpha ⇉\beta }=\min\left\{w_{ij}^{\alpha },w_{ij}^{\beta }\right\}$ is the multiplexed component of the weights associated with the links between *i* and *j* in layers α and β. In particular, $s_{i}^{\alpha ⇉\beta }$ is the multiplex quantity allowing us to describe the inter-layer weighted coupling. Figure 3 reports the relation between $s_{i}^{\beta }$ and $s_{i}^{\alpha ⇉\beta }$ for representative pairs of commodities of the WTM. We see a clear almost linear empirical trend, that can be approximated as:

(52) $$s_{i}^{\alpha ⇉\beta }\approx U^{\alpha \beta }s_{i}^{\beta }.$$

Our goal will consist of designing the minimal model able to capture this empirical evidence.

In this perspective, we define the corresponding expected quantities $\langle w_{ij}^{\alpha ⇉\beta }\rangle$ and $\langle w_{ij}^{\alpha }\rangle$. In particular, the multiplexed component can be written in terms of a joint probability in order to keep the same structure adopted for the binary case:

(53) $$\begin{array}{ccc} \langle w_{ij}^{\alpha ⇉\beta }\rangle & = & \;\langle \min\left\{w_{ij}^{\alpha },w_{ij}^{\beta }\right\}\rangle \\ & = & \;\sum_{w=1}^{\infty }P\left(\min\{w_{ij}^{\alpha },w_{ij}^{\beta }\}\geq w\right)\\ & = & \;\sum_{w=1}^{\infty }P\left(w_{ij}^{\alpha }\geq w\cap w_{ij}^{\beta }\geq w\right)\\ & = & \;\sum_{w=1}^{\infty }\;U_{ij}^{\alpha \beta }\;\left(w_{ij}^{\alpha }\geq w|w_{ij}^{\beta }\geq w\right)P\left(w_{ij}^{\beta }\geq w\right), \end{array}$$

where $U_{ij}^{\alpha \beta }$ is now the probability of observing a weight $w_{ij}^{\alpha }$ in α larger than *w* given that a weight $w_{ij}^{\beta }$ larger than *w* has been observed in β.

As mentioned, the phenomenological observation shows that the conditional probability defined in (53) is actually independent of the considered pair of nodes:

(54) $$\begin{array}{c} U_{ij}^{\alpha \beta }=\frac{\langle \min\left\{w_{ij}^{\alpha },w_{ij}^{\beta }\right\}\rangle }{\langle w_{ij}^{\beta }\rangle }\approx U^{\alpha \beta }. \end{array}$$

Applying the same transformations i↦j and α↦β, we get

(55) $$\begin{array}{ccc} U^{\alpha \beta }\langle w_{ij}^{\beta }\rangle & \approx & \langle \min\left\{w_{ij}^{\alpha },w_{ij}^{\beta }\right\}\rangle \\ & = & \langle \min\left\{w_{ij}^{\beta },w_{ij}^{\alpha }\right\}\rangle \\ & \approx & U^{\beta \alpha }\langle w_{ij}^{\alpha }\rangle . \end{array}$$

Summing Equation (55) over *i* and *j*, we obtain

(56) $$\begin{array}{c} U^{\alpha \beta }W^{\beta }=U^{\beta \alpha }W^{\alpha }. \end{array}$$

Similarly, inverting (54) we get

(57) $$\begin{array}{c} U^{\alpha \beta }\langle w_{ij}^{\beta }\rangle \approx \langle \min\left\{w_{ij}^{\alpha },w_{ij}^{\beta }\right\}\rangle \end{array}$$

and, summing over pairs of nodes, we obtain, as in the binary case,

(58) $$\begin{array}{ccc} U^{\alpha \beta } & = & \frac{\sum_{i}\sum_{j<i}\langle \min\left\{w_{ij}^{\alpha },w_{ij}^{\beta }\right\}\rangle }{\sum_{i}\sum_{j<i}\langle w_{ij}^{\beta }\rangle }\\ & = & \frac{\sum_{i}\sum_{j<i}\min\left\{w_{ij}^{\alpha },w_{ij}^{\beta }\right\}}{W^{\beta }}. \end{array}$$

We therefore arrive at

(59) $$\begin{array}{c} \frac{2}{m_{w}^{\alpha \beta }}=\frac{2\left(W^{\alpha }+W^{\beta }\right)}{2\sum_{i}\sum_{j<i}\min\left\{w_{ij}^{\alpha },w_{ij}^{\beta }\right\}}=\frac{1}{U^{\alpha \beta }}+\frac{1}{U^{\beta \alpha }}, \end{array}$$

where $m_{w}^{\alpha \beta }$ represents the entry of the weighted multiplexity matrix and $U^{\alpha \beta }$ is derived from the empirical relationship between $s_{i}^{\beta }$ and $s_{i}^{\alpha ⇉\beta }$. In analogy with the binary case, $m_{w}^{\alpha \beta }$ is therefore the harmonic mean of the conditional probabilities $U^{\alpha \beta }$ and $U^{\beta \alpha }$, as previously defined. Applying (56) to the previous expression, we get

(60) $$\begin{array}{ccc} \frac{2}{m_{w}^{\alpha \beta }} & = & \frac{1}{U^{\alpha \beta }}\left(1+\frac{U^{\alpha \beta }}{U^{\beta \alpha }}\right)\\ & = & \frac{1}{U^{\alpha \beta }}\left(1+\frac{W^{\alpha }}{W^{\beta }}\right)\\ & = & \frac{W^{\alpha }+W^{\beta }}{U^{\alpha \beta }W^{\beta }}. \end{array}$$

Thus, the value of the slope in the plots $s_{i}^{\alpha ⇉\beta }$ vs. $s_{i}^{\beta }$ should be, in the weighted case,

(61) $$\begin{array}{c} U^{\alpha \beta }=\frac{W^{\alpha }+W^{\beta }}{2W^{\beta }}m_{w}^{\alpha \beta }, \end{array}$$

in perfect analogy with the unweighted case. Indeed, in Figure 3 we show the comparison between the actual fit lines and the expected ones according to (61): the agreement is clear and robust across different pairs of commodities.

Therefore, in analogy with the unweighted case, here the minimal model suitable to reproduce the observed values of pairwise weighted multiplexity is based on the total multiplexed weight $W^{\alpha ⇉\beta }$ for any ordered pair of layers (α,β), accompanied by the strength sequences measured in any layer. We indeed show that any model that does not take into account some sort of weighted coupling between layers would not be sufficient, as shown by the results provided by the uncorrelated model (Figure 3).

### 4.2. Directed Weighted Model

Also, in the weighted case, it is possible to extend the analysis to the directed case. Here, the main goal consists of studying the relation between single-layer metrics and inter-layer weighted quantities, in order to model them by exploiting the notions of directed multiplexity and multireciprocity introduced before.

We have to define two distinct strengths, namely the out-strength

(62) $$\begin{array}{c} s_{i}^{\alpha ,out}=\sum_{j\neq i}w_{ij}^{\alpha } \end{array}$$

and the in-strength

(63) $$\begin{array}{c} s_{i}^{\alpha ,in}=\sum_{j\neq i}w_{ji}^{\alpha }. \end{array}$$

Moreover, the multiplex quantities will also split into two separate metrics, i.e., the *multiplexed strength*

(64) $$\begin{array}{c} s_{i}^{\alpha ⇉\beta }=\sum_{j\neq i}w_{ij}^{\alpha ⇉\beta }\equiv \sum_{j\neq i}\min\left\{w_{ij}^{\alpha },w_{ij}^{\beta }\right\} \end{array}$$

and the *multireciprocated strength*

(65) $$\begin{array}{c} s_{i}^{\alpha ⇄\beta }=\sum_{j\neq i}w_{ij}^{\alpha ⇄\beta }\equiv \sum_{j\neq i}\min\left\{w_{ij}^{\alpha },w_{ji}^{\beta }\right\}, \end{array}$$

where $w_{ij}^{\alpha ⇉\beta }=\min\left\{w_{ij}^{\alpha },w_{ij}^{\beta }\right\}$ is the multiplexed component of the weights associated with the directed links from *i* to *j* in layers α and β, and $w_{ij}^{\alpha ⇄\beta }=\min\left\{w_{ij}^{\alpha },w_{ji}^{\beta }\right\}$ is the reciprocated component. $s_{i}^{\alpha ⇉\beta }$ and $s_{i}^{\alpha ⇄\beta }$ are the metrics that will allow us to analyze and model the inter-layer coupling of the weighted WTM.

**Figure 4:**
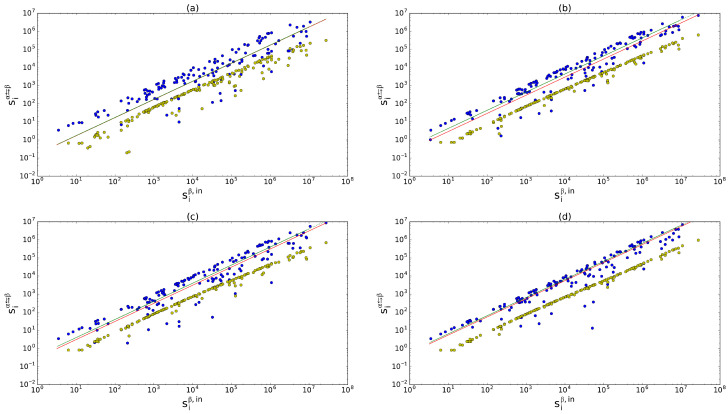
In-strength $s_{i}^{\beta ,in}$ (where β is the layer of trade in cereals) versus inter-layer multireciprocated strength $s_{i}^{\alpha ⇄\beta }$ against four other layers α: inorganic chemicals (**a**), plastics (**b**), iron and steel (**c**), and electric machinery (**d**). Blue dots: real data, which approximately follow the empirical linear relation in Equation (67); yellow dots: expected multireciprocated strength according to the uncorrelated model; lower green line: expected trend according to the theoretical slope $V^{\alpha \beta }$ in Equation (69); upper red line (when discernible): best fit. In all the cases, $R^{2}>0.95$ for both of the curves. It should be noted that we fit the empirical data with lines of the form y=a·x and only after we plot the results in log–log scale.

We empirically observe that the relations between $s_{i}^{\alpha ,out}$ and $s_{i}^{\alpha ⇉\beta }$ and between $s_{i}^{\alpha ,in}$ and $s_{i}^{\alpha ⇄\beta }$ (shown in Figure 4 for representative pairs of layers) are both approximately linear. Hence

(66) $$s_{i}^{\alpha ⇉\beta }\approx U^{\alpha \beta }s_{i}^{\beta ,out}$$

and

(67) $$s_{i}^{\alpha ⇄\beta }\approx V^{\alpha \beta }s_{i}^{\beta ,in}.$$

With the same reasoning developed for the undirected case, it is possible to derive the expected value of the prefactors $U^{\alpha \beta }$ and $V^{\alpha \beta }$, exploiting the notion of conditional probability. We determine that the model predicts

(68) $$\begin{array}{c} U^{\alpha \beta }=\frac{W^{\alpha }+W^{\beta }}{2W^{\beta }}m_{w}^{\alpha \beta } \end{array}$$

and

(69) $$\begin{array}{c} V^{\alpha \beta }=\frac{W^{\alpha }+W^{\beta }}{2W^{\beta }}r_{w}^{\alpha \beta }, \end{array}$$

where $m_{w}^{\alpha \beta }$ and $r_{w}^{\alpha \beta }$ are the corresponding entries of the multiplexity and multireciprocity matrices, respectively. The results of the fit of the model to the WTM are shown in Figure 4. The model is able to satisfactorily reproduce the values of multireciprocated strengths starting from the single-layer in-strengths (similar results are obtained for the relation between the out-strength and the multiplexed strength), while an uncorrelated model (i.e., without introducing any sort of dependency between layers) cannot capture the phenomenological observation.

Hence, in the weighted directed case, the most inexpensive reconstruction model builds on the knowledge of the in- and out-strength sequence of the different layers plus the M×M multiplexity and multireciprocity matrices.

## 5. Conclusions

The reconstruction of higher-order topological properties in multi-layer networks from single-layer information is an important problem which generalizes the more common problem of retrieving information from partial knowledge within a single-layer network. Indeed, in the multiplex case, the limitedness of information may affect multiple layers, but at the same time, the presence of correlations between different layers can be exploited to provide new routes to inference. Hence, any tool allowing us to infer inter-layer node-specific properties from the known information related to some particular layer is both theoretically interesting and practically useful. In this article we have provided a possible approach to the solution via the use of *multiplexed* and *multireciprocated degrees and strengths*, directly stemming from the previously defined *multiplexity* and *multireciprocity*. Our reconstruction technique builds on methods that have been shown to be well-grounded in the single-layer case. Indeed, previous studies highlighted that it is possible to correctly reproduce the topological structure of real-world graphs starting from limited information about, for instance, the strengths and the density of the considered system. Our methodology works for both binary and weighted networks and is able to also take into account the potential directionality of the links.

We must however stress that our technique is successfully applicable to systems exhibiting two main features. First, it should be possible to reliably reconstruct each single layer from the (approximate) knowledge of the strengths and/or the degrees of all nodes. Reconstruction methods that start from the *exact* knowledge of these local properties are, in the binary case, the Configuration Model [48,49] and the renormalizable Multi-Scale Model in [31] and, in the weighted case, the Enhanced Configuration Model [20,40,49]. Models that are instead based on *approximate* proxies of the degrees, derived from the overall link density and either the node strengths [21,22,23,24,29] or other features such as GDP [28,30,44,50,51], are also available. Economic and financial networks are found to fall into the category of systems that can be reliably reconstructed from the methods mentioned above [25,26]. Other systems, including some social [59] and biological [60,61] networks, have also been found to satisfy the required constraints and can therefore be successfully reconstructed through the presented techniques. Second, the conditional probabilities of observing a link in any layer, given that a link exists between the same pair of nodes in a different layer, should be independent of the considered nodes: in other words, such *multiplexity and multireciprocity probabilities* should be the same for all nodes and depend only on the pair of layers one is focusing on. Although the combination of the two assumptions may significantly restrict the range of systems that can be successfully reconstructed through our method, we have shown that one of most studied economic networks, namely the WTM, belongs to this class of reconstructable multi-layer networks. In general, we have shown that the measures of multiplexed or multireciprocated degrees and strengths can give very useful information about the coupling between layers. We have indeed explained that, by means of the aforementioned quantities, it is possible to acquire more refined notions of inter-layer coupling; multiplexed and multireciprocated degrees and strengths can therefore be thought of as new measures of multiplex assortativity, expressing the coupling caused by dependencies different to the simple correlation between degrees and/or strengths within and across layers.

## Data Availability

The world trade data analyzed in the article are freely available at refs. [46,47].
